# Complex chromosomal rearrangements induced by transposons in maize

**DOI:** 10.1093/genetics/iyac124

**Published:** 2022-09-16

**Authors:** Sharu Paul Sharma, Thomas Peterson

**Affiliations:** Department of Genetics, Development and Cell Biology, Iowa State University, Ames, IA 50011, USA; Department of Genetics, Development and Cell Biology, Iowa State University, Ames, IA 50011, USA; Department of Agronomy, Iowa State University, Ames, IA 50011, USA

**Keywords:** enhancer, RET, complex rearrangement, composite insertion, transposable elements, macrotransposons, Plant Genetics and Genomics

## Abstract

Eukaryotic genomes are large and complex, and gene expression can be affected by multiple regulatory elements and their positions within the dynamic chromatin architecture. Transposable elements are known to play important roles in genome evolution, yet questions remain as to how transposable elements alter genome structure and affect gene expression. Previous studies have shown that genome rearrangements can be induced by Reversed Ends Transposition involving termini of *Activator* and related transposable elements in maize and other plants. Here, we show that complex alleles can be formed by the rapid and progressive accumulation of *Activator-*induced duplications and rearrangements. The *p1* gene enhancer in maize can induce ectopic expression of the nearby *p2* gene in pericarp tissue when placed near it via different structural rearrangements. By screening for *p2* expression, we identified and studied 5 cases in which multiple sequential transposition events occurred and increased the *p1* enhancer copy number. We see active *p2* expression due to multiple copies of the *p1* enhancer present near *p2* in all 5 cases. The *p1* enhancer effects are confirmed by the observation that loss of *p2* expression is correlated with transposition-induced excision of the *p1* enhancers. We also performed a targeted Chromosome Conformation Capture experiment to test the physical interaction between the *p1* enhancer and *p2* promoter region. Together, our results show that transposon-induced rearrangements can accumulate rapidly and progressively increase genetic variation important for genomic evolution.

## Teaser

Using a convenient red kernel marker, Sharma and Peterson isolate a series of maize alleles containing Complex Chromosomal Rearrangements induced by the Ac transposable element. They show that these alleles were generated by the rapid and progressive accumulation of gene rearrangements that change the number and position of a known enhancer sequence. Their results demonstrate the remarkable capability of TEs to rapidly change genome structure and alter gene expression.

## Introduction

Enhancers are *cis*-regulatory DNA sequences that interact with their target promoters to stimulate transcription. These elements work independently of orientation and can be present near the promoter they influence, or they could act from large distances. Enhancers can be promiscuous or promoter-specific, affecting genes/promoters nearby, or passing over some genes to affect 1 further away (Kvon *et al.* 2014). With the discovery of *cis* elements working from a distance, it became clear that the functionality of the genome is not only dependent on the linear DNA sequence but also on the spatial arrangement of the chromatin (Doğan and Liu 2018; Krumm and Duan 2019). The eukaryotic nucleus has chromatin grouped into compartments of higher and lower transcriptional activity, called A and B compartments, respectively (Lieberman-Aiden *et al.* 2009). Furthermore, chromatin topologically associated domains (TADs) with boundaries controlled by cohesin and CCCTC-binding factor (CTCF) play important roles in the regulation of gene expression (Dekker *et al.* 2013; Nora *et al.* 2013). CTCF is conserved within bilaterian phyla, whereas plants lack CTCF or an ortholog (Heger *et al.* 2012). Hi-C maps do show compartmentalization and TADs in most plant genomes tested (Doğan and Liu 2018). Enhancers function within these domains, and structural rearrangements that change the TAD boundaries or position of an enhancer relative to their target genes can lead to dysregulation and disease (Lupiáñez *et al.* 2015; Bompadre and Andrey 2019). With only a few well-studied examples of enhancers in plants (Weber *et al.* 2016), little is known about the mechanism of their interactions with target promoters.

The maize *p1* and *p2* genes encode Myb-homologous regulators of the flavonoid biosynthetic pathway to produce red phlobaphene pigments in floral organs (Dooner *et al.* 1991; Grotewold *et al.* 1994). The *p1* gene is responsible for pigmentation in kernel pericarp, cob, and silk, while *p2* is expressed in anther and silk (Zhang *et al.* 2000; Goettel and Messing 2009). The striking red kernel phenotype specified by *p1* alleles has been used as a convenient indicator of gene expression since the earliest genetical studies in maize (Emerson 1917). The *p1* gene was one of the first loci shown to carry *Activator* (*Ac*) transposable element insertions (Barclay and Brink 1954; Greenblatt and Brink 1962; Lechelt *et al.* 1989). The presence of 1.2-kb enhancer region near the *p1* gene was inferred from early *Ac* insertional mutagenesis studies in the allele *P1-rr4B2* (Athma *et al.* 1992; Moreno *et al.* 1992). The enhancer activity of this region was confirmed by both transient and stable transformations using the *GUS* reporter gene (Sidorenko *et al.* 1999; Sidorenko *et al.* 2000). In addition to increasing the rate of transcription, the *p1* enhancer sequence has been shown to participate in *p1* paramutation (Sidorenko and Peterson 2001). The *P1-rr4B2* allele has a direct duplication of 1,269-bp fragment, which is a part of larger 5.2-kb direct repeats, which flank the *p1* gene. The 3′ 5.2-kb direct repeat contains 2 copies of the 1.2-kb repeat, whereas the 5′ 5.2-kb direct repeat contains 1 full-length 1.2-kb direct repeat, and a stable *Ds*-like element inserted into the upstream copy. The *p1* enhancer is a 405-bp fragment (fragment 15), which lies within the 1,269-bp direct duplication (Zhang and Peterson 2005a). For simplicity, following text refers to the 2 copies of fragment 15 as a single enhancer and the figures reflect the duplicate nature of the enhancer.

The maize *P1-rr11* allele has insertions of *Ac* and *fAc* (*fractured-Ac*) elements in the *p1* gene and produces a red pericarp phenotype. A derivative null allele *p1-wwB54* has white pericarp and white cob, and contains a deletion of the upstream enhancer and exons 1 and 2 of the *p1* gene (Yu *et al.* 2011). The *p1-wwB54* allele retains *p1* exon 3, the downstream enhancer, and the *Ac* and *fAc* elements in reversed orientation, separated by only 331 bp of DNA. These *Ac*/*fAc* termini can undergo frequent Reversed Ends Transposition (RET) events (Zhang and Peterson 2004; Huang and Dooner 2008; Zhang *et al.* 2009; Yu *et al.* 2011), forming a variety of alleles including deletions (Zhang and Peterson 2005b; Zhang *et al.* 2006), duplications (Zhang *et al.* 2013), Composite Insertions (*CIs*; Zhang *et al.* 2014; Su *et al.* 2018, 2020), and inversions (Zhang and Peterson 2004; Yu *et al.* 2011; Sharma *et al.* 2021). Here, we show how repeated RET events in maize can induce the formation of complex gene structures containing multiple rearrangements such as inversions, *CIs*, duplications and/or deletions. Moreover, these new structures alter the number and position of *p1* enhancer elements, thereby affecting expression of the neighboring *p2* gene.

## Methods

### Genetic screening and PCR

Nearly 4,000 plants of genotype *p1-wwB54* heterozygous with the null allele *p1-ww[4Co63]* were grown and pollinated with *p1-ww[4Co63]*. Ears were screened for kernels with red pericarp to obtain RET-induced rearrangement alleles. In *p1-wwB54*, we see approximately 1 in 8 ears with a single red kernel and 1 in 40 ears with a multikernel red sector. All 5 of the cases presented here were derived from single kernel events. Pericarp is a maternal tissue derived from the ovary wall, sharing a common lineage with the egg progenitor cells. The size and heritability of red pericarp sectors depend on the stage of ear and kernel development at which an activating mutation occurred. Mutations occurring earlier in development produce larger sectors, and the underlying mutation can be more frequently transmitted. Because recovery of any mutant allele will depend on the segregation of that allele to the egg cell at meiosis, only half of the events seen as single kernel sectors will be transmitted to the next generation (Anderson and Brink 1952). Red kernels were selected and planted, and in case of transmission, the resulting plants would produce whole red ears. Kernels from these red ears were sown, and genomic DNA was extracted from seedling leaves by a modified CTAB method (Saghai-Maroof *et al.* 1984). To detect structural rearrangements (Su *et al.* 2020; Sharma *et al.* 2021), PCR was performed under standard conditions using Promega GoTaq Green Master Mix and primers specifically designed for the *p1-wwB54* sequence (Supplementary Table 1). The models of RET predict that in rearrangements arising from the activity of *Ac* and *fAc* elements, each breakpoint will be expected to border a transposon involved in the reaction. *Ac* casting (Singh *et al.* 2003; Wang and Peterson 2013) and inverse-PCR (iPCR; Ochman *et al.* 1988) techniques were used to locate these breakpoints adjacent to *Ac* and *fAc*, respectively. For visualizing PCR products, a high-efficiency agarose gel electrophoresis method was used (Sharma and Peterson 2021). PCR amplicons were sequenced by the Iowa State University DNA Sequencing Facility.

### Southern blotting

To confirm the internal structure of the complex rearrangements, digests using *Bgl*II, *Kpn*I, *Hpa*I, and *Eco*RI restriction enzymes and their combinations were performed (data not shown for *Eco*RV and some combinations). For double digests, digestions were performed in 2 steps, 1 enzyme at a time. Whole-genome DNA was digested and loaded on an agarose gel (0.7–0.8%) run for 24–26 h under 35–40 V for adequate separation of fragments. The DNA was transferred to a membrane for 24 h, followed by probing the membranes with fragment 15 located within the *p1* gene enhancer (Zhang and Peterson 2005a).

### RT-PCR

Pericarps were peeled from kernels 20 days after pollination (DAP) and flash-frozen in liquid nitrogen. Two biological replicates (pericarps from 2 siblings) were pooled to extract RNA. RNA was isolated using Purelink Plant RNA Reagent, treated with New England Biolabs DNase I to remove gDNA, and then reverse transcribed to cDNA using Invitrogen SuperScript II Reverse Transcriptase kit. Protocols recommended by the product suppliers were used. Two technical replicates were used for each sample in reverse transcription reaction. Finally, PCR was performed on the cDNA using primers specific to exons 1 and 3 of *p2* to amplify the *p2* gene transcript (Supplementary Table 2). Primers specific to the *GAPDH* gene were used as an internal control (Supplementary Table 2).

### Chromosome conformation capture

A plant-specific 3C protocol was used with modifications (Louwers *et al.* 2009). Pericarps were peeled on ice from _**∼**_40 developing kernels at 15 DAP totaling approximately 700 mg of tissue and added directly to a 2% formaldehyde solution made with a nuclei isolation buffer. The pericarps were fixed at room temperature for 1 h in a vacuum chamber under 11.7 psi pressure. For digestion, the sample was equally divided into 2 tubes, 150 units of *Bgl*II were added to each and kept overnight at 37°C while on rotation. An additional 50 U of *Bgl*II were added to each tube the next morning and incubated for 2 h. Slow rotations of 60 rpm were used on all steps requiring rotation. For DNA precipitation, samples were stored at −20°C. *S-adenosyl-methionine decarboxylase* (*Sam*) gene was used as an internal control in qPCR. As a control for primer efficiency differences in PCR amplification, the target regions were amplified and digested with *Bgl*II and re-ligated (Tolhuis *et al.* 2002). The DNA concentration was determined and mixed in equimolar amounts to make the control template containing all possible ligation products of the loci of interest (*p1/p2* and *Sam*). qPCR was performed using SybrGreen master mix and supplier recommended protocols.

## Results

Southern blot analysis using fragment 15 (Zhang and Peterson 2005a) allowed us to identify alleles with multiple *p1* enhancers (Supplementary Fig. 1). In these alleles, rearrangement endpoints were found using *Ac* casting (Singh *et al.* 2003; Wang and Peterson 2013) and/or inverse-PCR (iPCR; Ochman *et al.* 1988) techniques. By evaluating the Southern blot results (Supplementary Figs. 1–3) together with the breakpoint sequences found with PCR techniques, the structures of 5 different rearrangement alleles were deduced in accordance with existing models of RET (Zhang and Peterson 2004; Huang and Dooner 2008; Zhang *et al.* 2009; Yu *et al.* 2011). The presence of 8-bp target site duplications (TSDs) at the 2 endpoints of each rearrangement structure confirmed their origin from *Ac* transposition (Supplementary Table S3). The structures of some cases could not be completely determined and thus are not considered here.

**Fig. 1.**
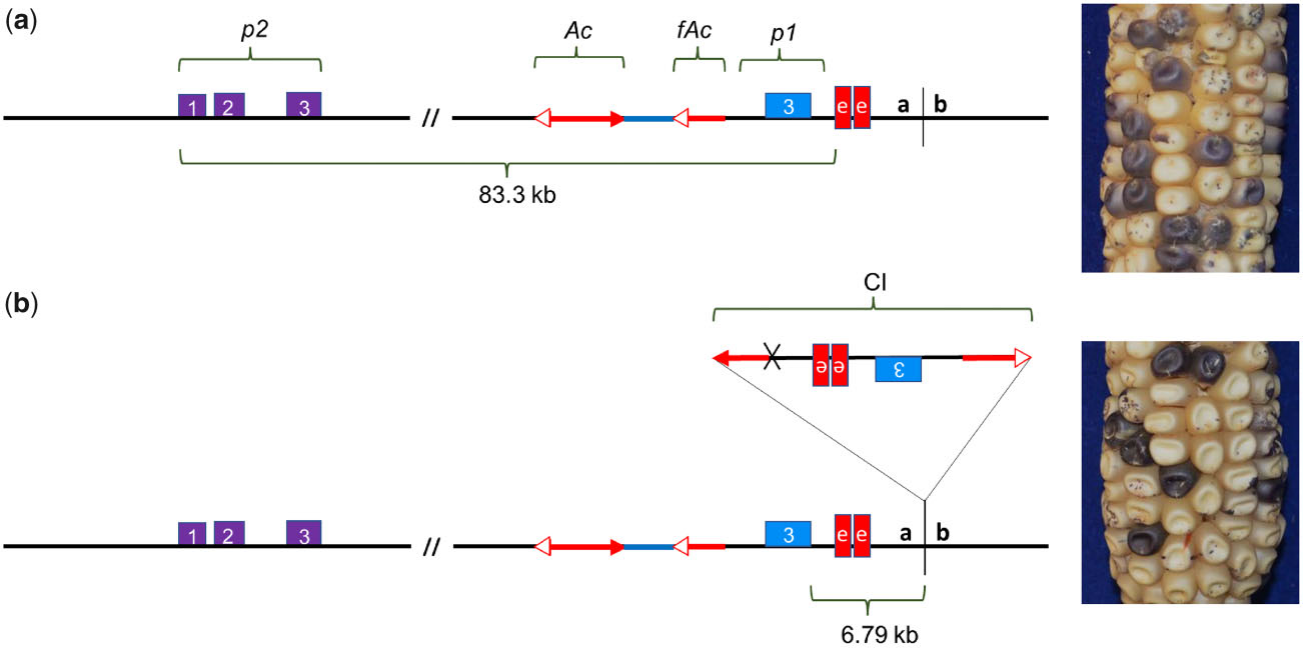
a) *p1-wwB54:* purple boxes represent the *p2* gene with numbered exons 1, 2, and 3. The blue box is exon 3 of the *p1* gene. Red boxes indicate 2 copies of fragment 15. Red arrows are *Ac* (2 arrowheads) and *fAc* (single arrowhead) elements. b) *p1-wwB54-CI* contains a *CI* at the site labeled *a/b* 6.79-kb downstream of *p1* exon 3. This *CI* contains 1 copy of the enhancer region and exon 3 and is also present in 5 other rearrangement alleles. Corresponding pictures on the right show colorless pericarp phenotype in both alleles. The solid and spotted purple color is due to *r1-m3::Ds* activation in aleurone caused by *Ac*-induced excision of *Ds*.

The null allele *p1-wwB54* has a white kernel pericarp phenotype, and the *p2* gene is not expressed in the pericarp (Sharma *et al.* 2021). The *p1-wwB54* allele retains the *p1* enhancer downstream of *p1* exon 3; as described above, there are 2 nearby copies of the fragment 15 enhancer, indicated by the 2 red boxes in Fig. 1a (Zhang and Peterson 2005a). Structural rearrangements that move the *p1* gene enhancer closer to the *p2* gene can induce expression of the *p2* gene in the pericarp and produce a red kernel pericarp phenotype. Secondary rearrangements are possible in many cases due to the continued presence of *Ac/fAc* termini and their ability to interact (Fig. 1a). Some of the rearrangements previously studied are deletions (Zhang *et al.* 2006), inversions (Sharma *et al.* 2021), and Composite Insertions (Su *et al.* 2020). The model for the formation of Composite Insertion (*CI*) through DNA re-replication has been described previously in detail (Zhang *et al.* 2014; Su *et al.* 2020). Briefly, a *CI* arises when a transposon pair along with flanking DNA moves during replication from an already replicated part to a nonreplicated region and gets re-replicated.

At some point during the propagation of *p1-wwB54*, a new *CI* inserted into a site 6.79_**-**_kb downstream of the *p1* exon 3 (Fig. 1, site *a/b*). This *CI* consists of 1,391 bp of *Ac* 5′ side sequence joined with 9,022 bp of *fAc* and flanking region containing *p1* exon 3 and the *p1* enhancer region. These 2 *CI* fragments are fused at a 4_**-**_bp sequence overlap (Supplementary Table 3), suggesting they were joined by microhomology-mediated end joining (McVey and Lee 2008). This *CI* insertion created a new allele termed *p1-wwB54-CI* (Fig. 1b), which contains 2 *p1* enhancers located at 83.35 and 92.4 kb from the *p2* 5′ end. Similar to *p1-wwB54*, the *p1-wwB54-CI* allele has colorless pericarp. The lack of *p2* expression in both alleles indicates that 1 or 2 copies of the enhancer cannot act on *p2* from this distance. Because the kernel pericarp phenotype of *p1-wwB54-CI* is not significantly different from the parental *p1-wwB54*, the presence of *p1-wwB54-CI* remained undetected in our materials until we characterized 5 independent derivative alleles. These alleles all contained a *CI* with the same internal structure, inserted at the same position (Fig. 1, site *a/b*), and flanked by the same 8-bp TSD as that of *p1-wwB54-CI* (Supplementary Table 3). We conclude that the 5 mutant alleles were all derived from *p1-wwB54-CI.* The 5 complex rearrangements describe here appeared within 5 maize generations since the first isolation of *p1-wwB54* (Yu *et al.* 2011).

### Structures of complex rearrangements

**Fig. 2.**
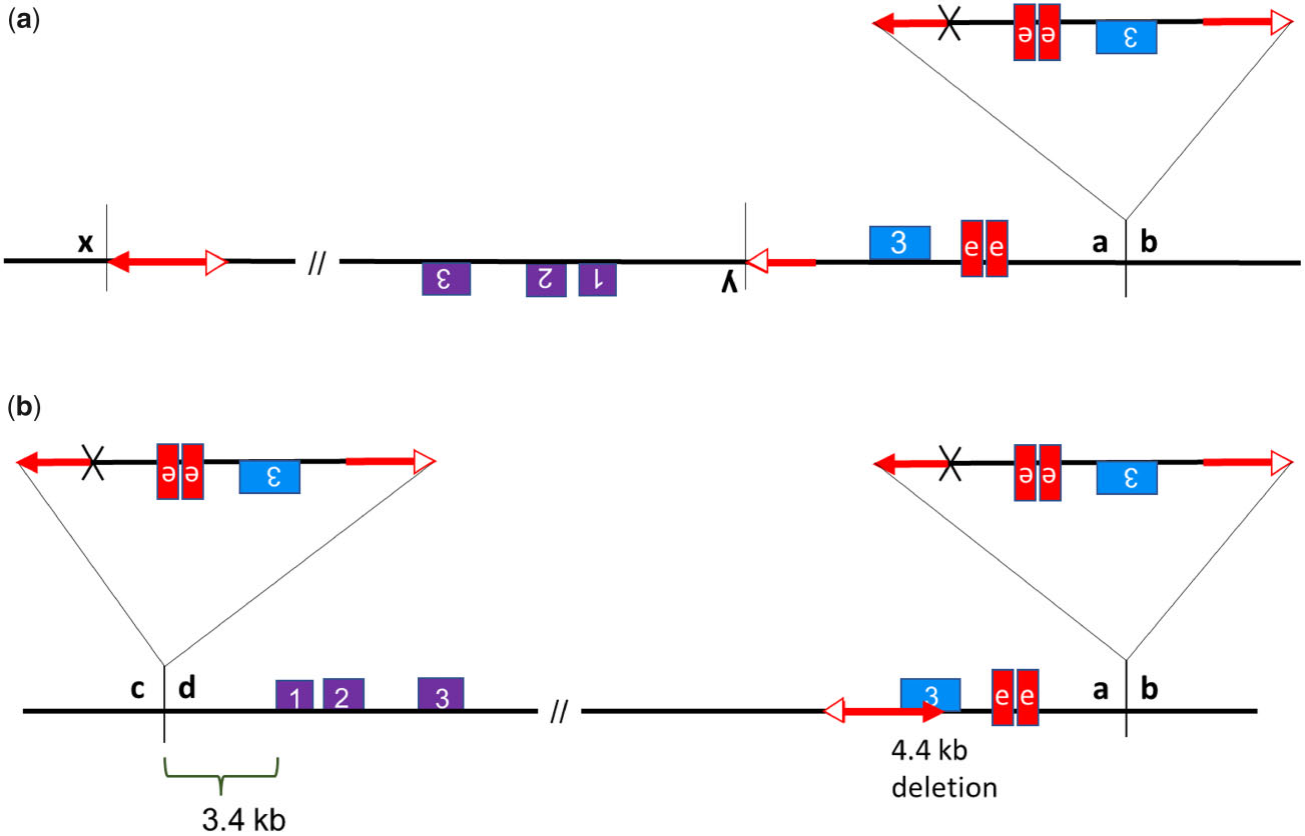
a) SP-6, *SP-7*, and *SP-12*: these alleles have unique *x/y* inversion endpoints and share the same *a/b* insertion site for the common *CI* containing a copy of the *p1* enhancer. b) *SP-11* has a copy of the same *CI* inserted near *p2* and an additional 4.4-kb deletion in *p1*.

**Fig. 3.**
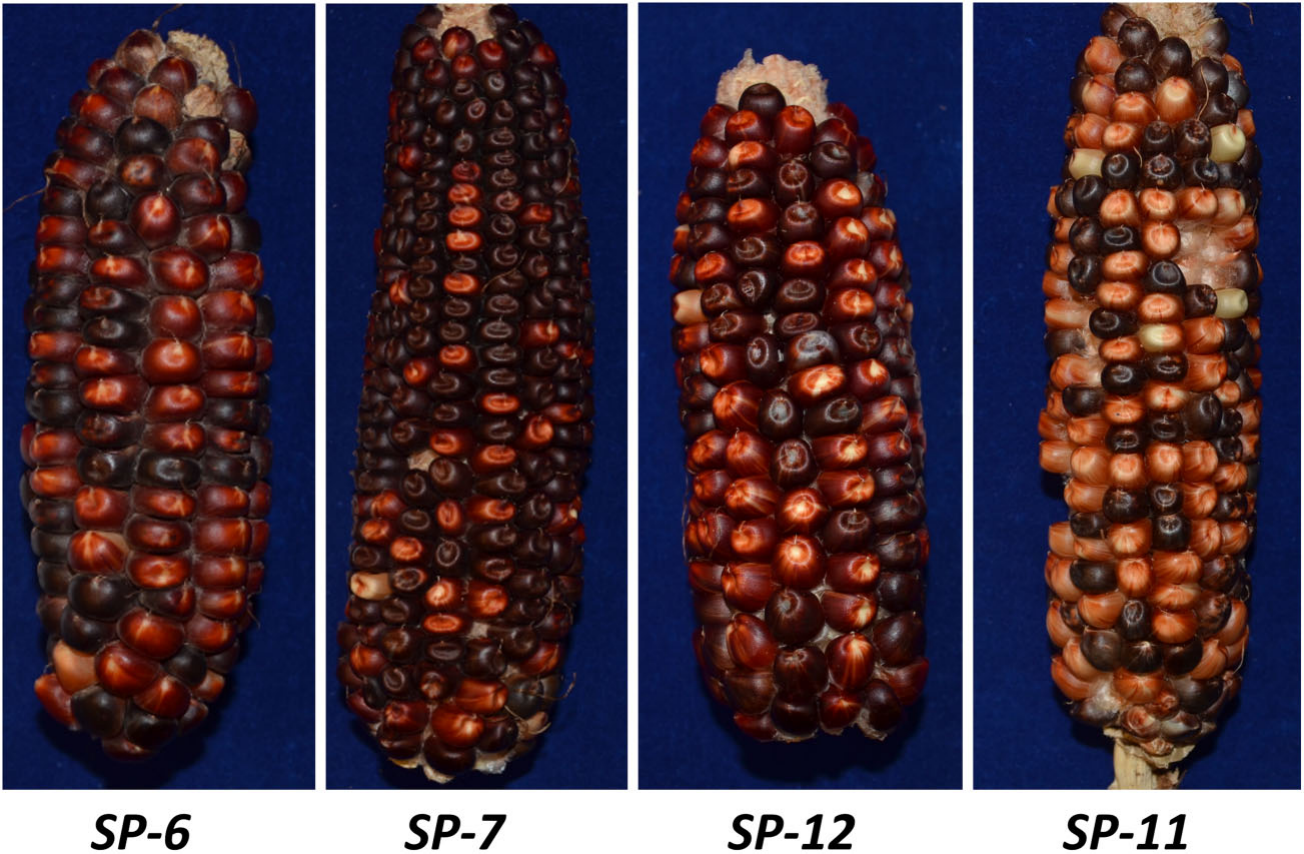
Ears of alleles with red pericarp phenotype. *SP-6*, *SP-7*, and *SP-12* have darker red pericarp phenotypes compared to *SP-11*. The dark purple color in some kernels is due to anthocyanin pigmentation of kernel aleurone induced by *Ds* excision from the *r1-m3::Ds* tester allele.

Five cases labeled SP-6, SP-7, SP-12, SP-11, and SP-97 with independent rearrangement structures were isolated. These cases contain combinations of rearrangements such as inversions, *CIs*, and deletions. All 5 cases share the same *CI* downstream of the *p1* enhancer with *p1-wwB54-CI*. In addition, *SP-6*, *SP-7*, and *SP-12* have an inversion, each with a unique endpoint near *p2* (Fig. 2a). The target site *x/y* in *SP-6*, *SP-7*, and *SP-12* is 199, 257, and 3,041 bp away from the *p2* transcription start site (TSS), respectively, making the distances between *p2* TSS and *p1* enhancers from 4.9 to 16.8 kb. Target site *x/y* in each case was found to have flanking 8_**-**_bp target site duplications (Supplementary Table S3). In allele *SP-6*, the rearrangement structure leaves only 207 bp of promoter region sequence upstream of *p2*. Compared to *p1-wwB54*-*CI*, the inversions reduced the distance between *p2* and the *p1* enhancers in these cases. We expect *SP-11* (Fig. 2b) to have originated from a progenitor that had the same structure as Fig. 1b. The *CI* structure with *Ac* and *fAc* terminal sequences at its ends is known to be able to transpose (Su *et al.* 2020). If the *CI* excises from its *p1* location after being replicated and then inserts into a yet unreplicated region, the resulting allele will retain 2 copies of the *CI*. *SP*-*11* has the common *CI* at the *p1* downstream site, and a copy of the same *CI* is present near *p2* (Fig. 2b). In addition to the movement/duplication of the *CI*, *SP-11* has a deletion toward *p1* exon 3, which could result from the RET event in which the *Ac/fAc* pair inserted into *p1* exon 3, leading to deletion of the 4.4_**-**_kb fragment containing *fAc* and part of *p1* exon 3 (Fig. 2b). With the 2 *CIs* carrying 1 copy of the *p1* enhancer each, *SP-11* has a total of 3 *p1* enhancers. The enhancer in the *CI* inserted near *p2* is at about 8.1 kb, and the 2 distant enhancers are at 78.95 and 88 kb from *p2*. *SP-6*, *SP-7*, and *SP-12* ears have a dark red pericarp phenotype, whereas the *SP-11* ear is fainter red in comparison (Fig. 3). The lighter red phenotype could possibly indicate that the 2 distant enhancers are not involved in *p2* activation in *SP-11* same as in *p1-wwB54-CI*. RT-PCR results show that these 4 alleles with red pericarp have active *p2* gene expression in pericarp tissue (Supplementary Fig. 4). The *p2* expression in pericarp was confirmed by sequence information (Sharma *et al.* 2021).

### Progressive rearrangements in *SP-97*

**Fig. 4.**
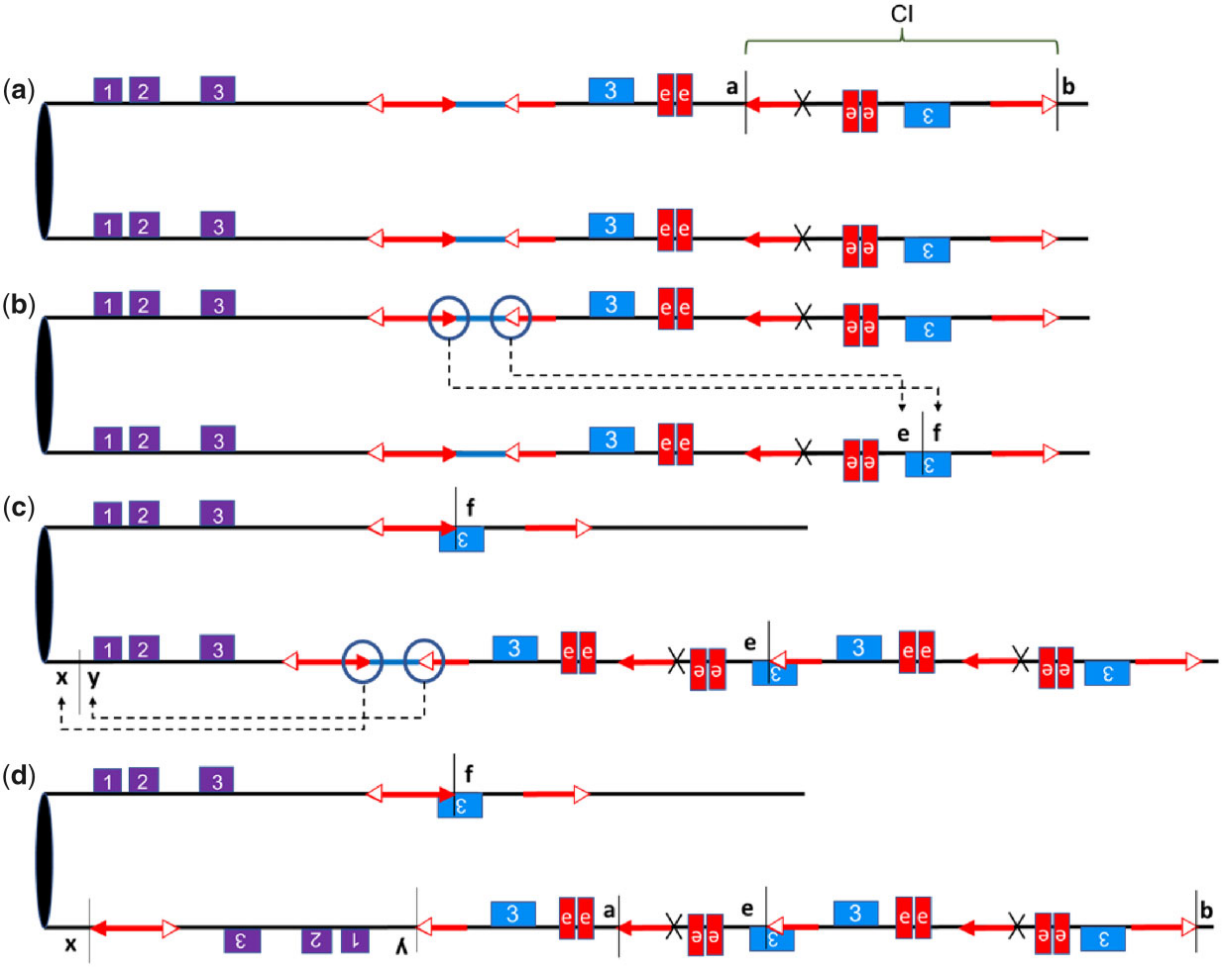
Model for the origin of allele *SP-97*: a) ancestral allele, which has a structure similar to *p1-wwB54-CI*. The diagram shows sister chromatids and the *CI* downstream of the *p1* enhancer at site *a/b*. b) RET: *Ac* and *fAc* pair move from 1 chromatid and insert into the *e/f* target site in the sister chromatid, causing duplication and reciprocal deletion in the sister chromatids. c) RET: The *Ac/fAc* pair on the other chromatid underwent an inversion toward *p2* inserting at target site *x/y*. d) The lower chromatid is *SP-97*, which contains 4 copies of the *p1* enhancer, an inversion, a *CI*, and a duplication.

The ability of multiple *Ac/fAc* elements to interact and transpose leaves many possibilities for the formation of different complicated structures. This potential is exemplified in the fifth allele *SP-97*, which has a complex structure with 4 *p1* enhancers and a dark red phenotype. Figure 4 describes a model for the origin of *SP-97*. We hypothesize that it originated after the common *CI* at the *a/b* site in *p1-wwB54* (Fig. 4a, same as Fig. 1b); the *Ac* and *fAc* elements present at their original position underwent reversed ends transposition into the sister chromatid at site *e/f* (Fig. 4b). This resulted in a tandem direct duplication of the 17.73_**-**_kb region containing 2 copies of the enhancer, bringing the total to 4 copies (Fig. 4c). The *Ac* and *fAc* elements in the recipient chromatid are also capable of generating rearrangements. A subsequent RET event with insertion of *Ac/fAc* pair at target site *x/y* 20.3 kb from *p2* caused an inversion that brought *p2* closer to the 4 enhancers leading to activation in the pericarp (Fig. 4, c and d). The size of the inverted fragment is 98.5 kb (*x* to *y* in Fig. 4d). The final structure in Fig. 4d lower chromatid is the *SP-97* allele containing a *CI*, a duplication, and an inversion. It would take these 3 RET events to form the final structure of *SP-97* leading to the red pericarp phenotype selected. The event consisting formation of the *CI* occurred prior to other 2 since it is present in other alleles as well. The duplication and inversion could have occurred later in a single or 2 different generations. The inversion would occur at last reducing the distance between the *p2* gene and the *p1* enhancers. To confirm this structure, endpoints *a/b* and *x/y* were sequenced and found to contain the flanking 8_**-**_bp target site duplications (Supplementary Table 3). The new junction created by the duplication at target site *e/f* (Fig. 4, b and c) was also sequenced (Supplementary Table 3). The distance of the 4 enhancers from *p2* is about 25, 34, 41.5, and 50.5 kb.

**Fig. 5.**
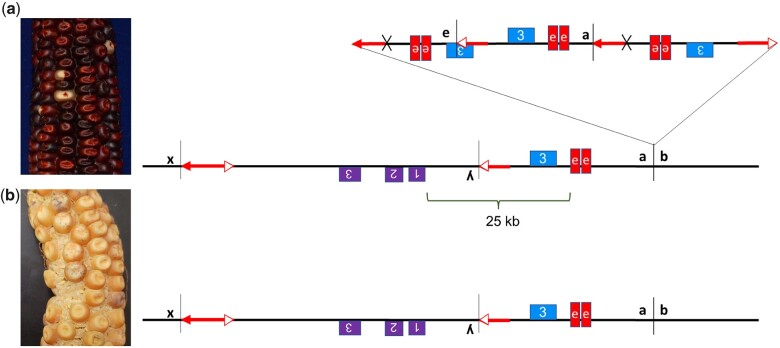
Phenotype (left) and structure (right). a) *SP-97* has a dark red kernel pericarp. The final structure of *SP-97* from Fig. 4 is consistent with a 28-kb macrotransposon at site *a/b*. b) *SP-97M1* has a light red kernel pericarp. The large macrotransposon excised out, leaving only 1 copy of the enhancer in the resulting allele at 25 kb from *p2*.

**Fig. 6.**
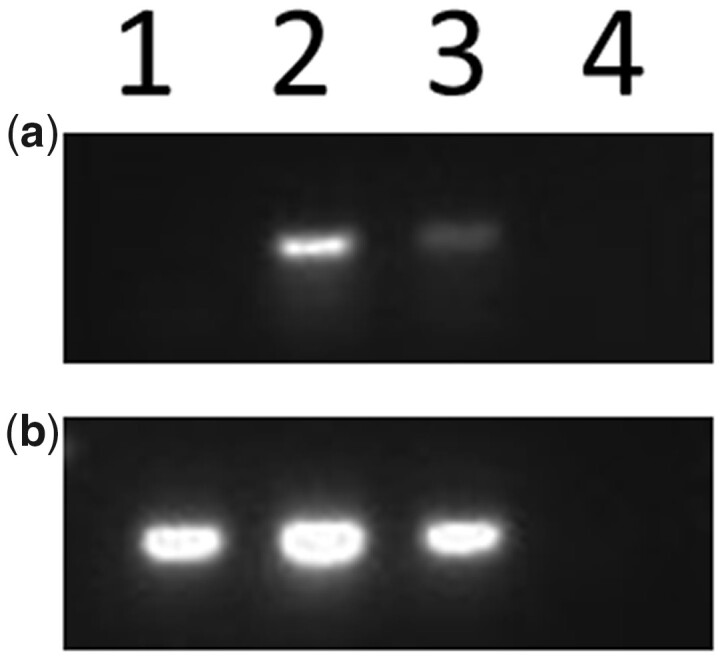
RT-PCR using RNA extracted from pericarp tissue and reverse transcribed to cDNA. Agarose gel image showing results of RT-PCR with primers from a) *p2* exons 1 and 3, b) GAPDH as an internal control. Lane 1, *p1-wwB54-CI*; lane 2, *SP-97*; lane 3, *SP-97M1*; lane 4, negative control. *p1-wwB54-CI* lacks *p2* expression; *SP-97* and *SP-97M1* have *p2* expression.

If the dark red phenotype results from multiple copies of the *p1* enhancer acting on *p2*, then the absence of some of these enhancers should affect the phenotype. To test this hypothesis, we examined ears produced by different alleles for the loss-of-function colorless kernels. *SP-97* had some colorless/faint red kernels on the ears with dark red kernel pericarp (Fig. 5a), which gave rise to the stable mutant called *SP-97M1*, which has a very faint red pericarp phenotype (Fig. 5b). *SP-97* has 4 copies of the *p1* enhancer near *p2*. Three of the 4 copies are within a structure that ends with *Ac* 5′ and 3′ terminal sequences, potentially forming a 28_**-**_kb large macrotransposon, which might be capable of transposition (Huang and Dooner 2008; Su *et al.* 2020). We analyzed the structure of *SP-97M1* and found that the entire macrotransposon structure containing 3 copies of the *p1* enhancer has been excised; the excision site retains a modified 8-bp TSD as a macrotransposon footprint (Supplementary Table S3). The resulting *SP-97M1* allele is left with only 1 copy of the *p1* enhancer at 25 kb from *p2*. *SP-97M1* still has some *p2* activity in the pericarp (Fig. 6). Similarly, loss-of-function mutants from *SP-7* and *SP-12* (called *SP-7M1* and *SP-12M1*, respectively) were also found to have a light red pericarp phenotype (Supplementary Fig. 5). In both cases, the *CI* excised out, leaving only a single copy of the *p1* enhancer near the *p2* gene. An 8-bp TSD footprint was also sequenced in *SP-7M1* (Supplementary Table 3). The lighter red phenotype in all 3 mutants shows that the darker red phenotype was a result of multiple copies of the enhancer present close to the *p2* gene.

### Enhancer–promoter interaction

**Fig. 7.**
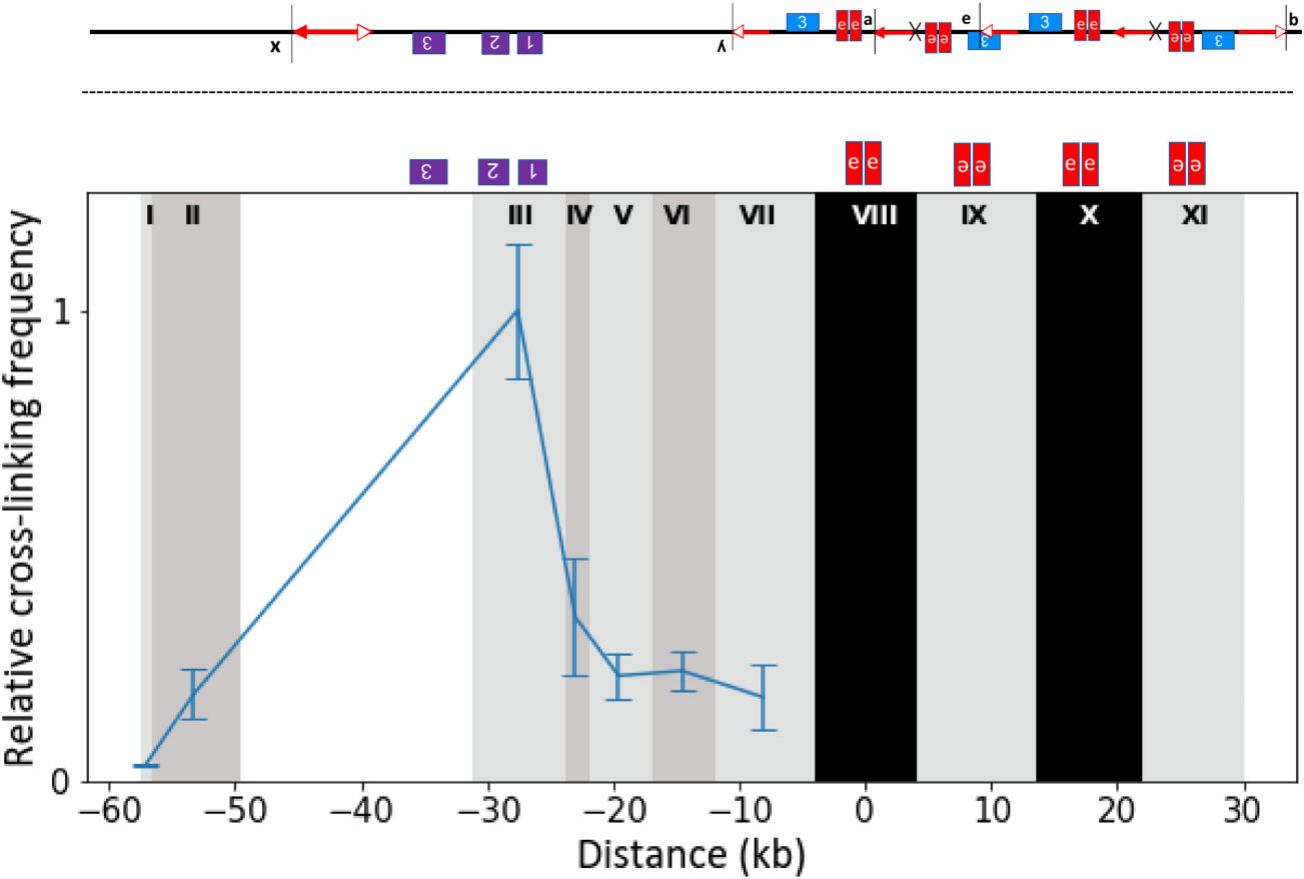
(Top) The structure of SP-97 from Fig. 4d. (Bottom) Relative cross-linking frequency at *p1/p2* locus in *SP-97*. The vertical shaded columns indicate the location of *Bgl*II fragments, numbered with Roman numerals. The anchor fragments are shaded in black. The blue line shows the relative crosslinking frequency of fragments tested against the anchors. Error bars indicate the standard error of the mean of 3 samples. The *y*-axis is cross-linking frequency, and the *x*-axis is the distance in kb. Location of *p1* enhancers and *p2* gene is shown above the graph. Fragments VIII and X have the same sequence and orientation, whereas fragments IX and XI are structurally different and in the opposite orientation. This is due to the duplication event in the origin of *SP-97* (Fig. 4, b and c).

If the *p1* enhancer is in fact activating *p2* expression in the pericarp, we may be able to detect physical interactions between the *p1* enhancer and *p2* promoter. We tested for such interactions in Chromosome Conformation Capture (3C) experiments (Louwers *et al.* 2009) performed in the *SP-97* allele, which was chosen for its dark red pericarp color. First, fresh pericarp tissue was cross-linked and intact nuclei were isolated. Then, the cross-linked nuclei were digested with *Bgl*II, the DNA re-ligated, and the crosslinks reversed. The resulting 3C DNA consists of new DNA molecules formed by the ligation of the *Bgl*II ends of DNA fragments from interacting loci. These new molecules were tested using primers specific to *Bgl*II fragments around *p1/p2* region (Supplementary Table 4). Due to the presence of many repetitive retroelement sequences inserted within a 100-kb region around *p2*, many *Bgl*II fragments could not be tested. We were able to test 7 fragments (Fig. 7, labeled I to VII) located within a _**∼**_60-kb region encompassing the *p2* gene for interactions with the *p1* enhancer. Due to the complex duplication in the *SP-97* allele, the *p1* enhancer is present on 4 *Bgl*II fragments labeled VIII to XI (Fig. 7). It is unknown whether all 4 copies of the *p1* enhancer may interact with *p2*, so we tested the interaction of the *p1* enhancer closest to *p2.* A primer specific to fragment VIII (and X) was used as an anchor and tested against primers specific to fragments I to VII. As shown in Fig. 7, the experiment detects a strong interaction peak in fragment III containing the *p2* promoter region and lower interaction frequency in other nearby fragments. This result suggests that the interaction is specific between the *p1* enhancer and the *p2* gene; if there were no specific interactions and only random passive interactions, 1 would expect the highest interaction frequency with the fragment nearest the enhancer (fragment VII) and a declining frequency at further distances. The comparison of 7 different fragments from both sides of the *p2* gene provides a level of internal control for the experiment. Additional 3C experiments testing other sites and alleles could further define the interaction, but such experiments are beyond the scope of this study.

## Discussion

### Progressive rearrangements form complex alleles

Complex Chromosomal Rearrangements (CCRs) are defined in the human genetics literature as structures consisting of more than 2 breakpoints and involving 2 or more chromosomes (Pellestor *et al.* 2011). CCRs are clinically important as they are involved in a number of abnormal phenotypes such as recurrent miscarriages (Giardino *et al.* 2009), mental retardation (Batanian and Eswara 1998), and congenital malformations (Vermeulen *et al.* 2004). In addition to disease-causing variation, CCRs are important for the formation of loci with adaptive benefits in both animals and plants. Fibrinogen locus in humans, which is a major clotting factor (Kant *et al.* 1985), and the *sh2-R* allele of the maize *shrunken-2* locus which gave rise to the sweetcorns (Kramer *et al.* 2015; Hu *et al.* 2021) are 2 examples of alleles originating from multiple rearrangements leading to a complex structure. The different *p2* alleles we present here are also examples of CCRs in plants, except for the involvement of a single chromosome in their formation. We show that a pair of DNA transposable elements can form CCRs by recurrent alternative transposition events, leading to the formation of complex alleles that can alter gene expression.

Previous studies have shown that alternative transposition of *Ac/Ds* elements can generate genomic rearrangements (Zhang *et al.* 2009; Yu *et al.* 2011). Even a single rearrangement event can have a dramatic effect on gene expression (Sharma *et al.* 2021). In this study, we used the red pericarp phenotype as an indicator of *p2* gene activation. We screened for red kernels to identify rearrangements caused by the movement of a pair of DNA elements (*Ac/fAc*). We show that these elements remain active and capable of undergoing transpositions causing progressive rearrangements. We identified CCRs with *p2* activity, but it is possible for other CCRs to occur without activation of *p2*. So, although we describe only 5 CCRs among 4,000 ears, the total number of CCRs is likely much higher. The 5 CCR alleles described here consist of multiple rearrangement events leading to the activation of the *p2* gene due to the presence of the *p1* enhancer in close proximity. In addition to the enhancer being able to interact with the target gene, the loss of function in *SP-97M1*, *SP-7M1*, and *SP-12M1* alleles with a decrease in the number of copies of the enhancer indicates that multiple enhancers work together to induce the comparatively darker red phenotype in *SP-97*, *SP-7*, and *SP-12*.

RET-induced DNA re-replication can generate *CIs* that act as *Ac*-macrotransposons (Zhang *et al.* 2014; Su *et al.* 2020). *Ac*-macrotransposons have been found in several different maize lines highlighting their role in genome evolution (Wang *et al.* 2022). The *SP-11* allele is an example of the reinsertion of a 10.3-kb *CI* containing the *p1* enhancer near the *p2* gene. The retention of the macrotransposon at its original location together with the insertion of a second copy shows that these macrotransposons can increase in number, as previously proposed for *Ac* element transposition (Greenblatt and Brink 1962; Chen *et al.* 1987). In addition to the 10.3-kb macrotransposon present in all the cases discussed in our results, we present the example of *SP-97*, which contains a large macrotransposon of 28-kb size. The mutant *SP-97M1* shows that this macrotransposon is able to excise. *Ac/fAc* termini in reverse or direct orientation have been known to cause chromosome breaks at a frequency inversely proportional to the distance between the interacting termini (Dooner and Belachew 1991; Yu *et al.* 2010). Both *SP-12* and *SP-97* were found capable of chromosome breakage, although breakage is much more frequent in *SP-97* (Supplementary Fig. 6), likely due to the presence of numerous closely spaced *Ac/fAc* termini.

### Effects of multiple enhancers

**Fig. 8.**
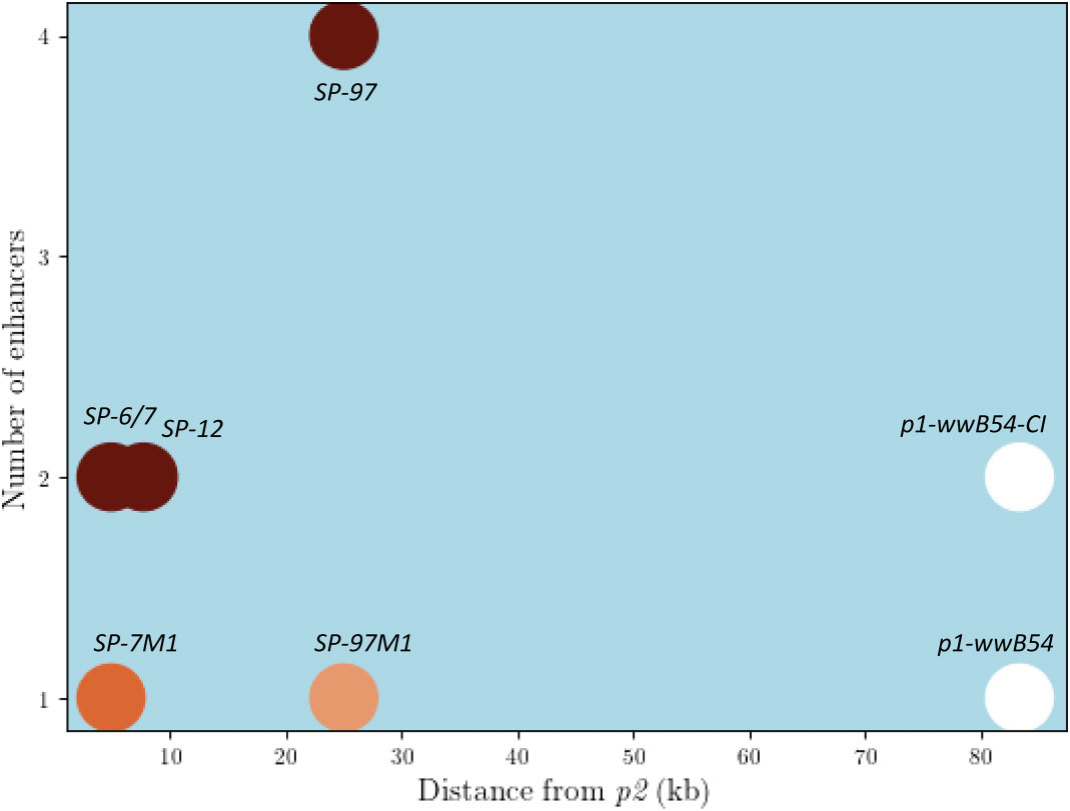
Relationship between enhancer number, distance from *p2*, and pericarp phenotype. The circles represent different alleles and their pericarp color, the *x*-axis is the distance of the nearest enhancer in kb from *p2*, and the *y*-axis is the number of enhancers. The darker red phenotype seems to have a positive correlation with the number of enhancers and a negative correlation with the distance from the target gene. Additional factors may affect intensity of pericarp color.

There is clearly a limited distance up to which the *p1* enhancer can influence *p2* gene expression. The 2 cases which have the *p1* enhancers more than 80 kb away from *p2*, the parental *p1-wwB54* allele with 1 *p1* enhancer and *p1-wwB54-CI* with 2 *p1* enhancers, both have colorless pericarp. But all alleles with active *p2* expression in the pericarp have at least 1 enhancer within 25 kb of *p2*, indicating that the distance at which a single enhancer can no longer interact with *p2* is somewhere between 25 and 80 kb. Among cases with a single *p1* enhancer, the pericarp color gets lighter with an increase in distance (Fig. 8), indicating that the interaction is distance dependent. A positive correlation between gene expression and enhancer proximity has also been reported at a genomic scale (Downes *et al.* 2021) and a single locus (Nolis *et al.* 2009). Although enhancers are known to work from large distances, these long-range interactions are enabled by facilitating mechanisms such as chromatin looping and transcription factors that help the enhancer to reach the target promoter (Deng *et al.* 2012; Bartman *et al.* 2016). In addition to these facilitating mechanisms, an enhancer might have an intrinsic range in which it can interact with its target promoter. In a 2009 study using transgenic HeLa cells, Nolis *et al.* show the IFN-β enhancer activates transcription only up to a distance of 560 bp but with the addition of binding sites for Sp1 and CCAAT enhancer binding protein transcription factors, the range increases to at least 2,325 bp. In our case, *SP-97M1* has a lighter red phenotype with the single enhancer at 25 kb, and *SP-97* has a darker red phenotype with additional enhancers at distances of 34, 41.5, and 50.5 kb, which are all larger than 25 kb (Figs. 4 and 5), suggesting that presence of multiple enhancers can increase the maximum distance at which productive enhancer–promoter interactions can occur. If increasing the enhancer number strengthens the enhancer–promoter interaction, it is possible that it could also disrupt the 3D chromatin structure (Chakraborty *et al.* 2022).

Long-range transcriptional *cis*-regulatory elements, including enhancers, are widespread in the maize genome (Ricci *et al.* 2019). A hepta-repeat present at 100-kb upstream from the *b1* gene and distal *cis*-element present at 140-kb upstream of the *BX1* gene are candidate enhancer sequences for their respective target genes. In both cases, alleles containing multiple copies of these sequences have higher expression compared to their single copy counterparts (Louwers *et al.* 2009; Zheng *et al.* 2015). Here, we show that alleles containing multiple copies of the known *p1* enhancer tend to exhibit darker red pericarp, while alleles with a single *p1* enhancer copy near *p2* specify lighter red phenotype (Fig. 8). Importantly, *SP-97* with 4 enhancers has significantly greater pericarp color than its single enhancer derivative *SP-97M1* (Fig. 6). A similar correlation between enhancer number and expression has been reported in transgenic grape and tobacco plants (Li *et al.* 2004). Having multiple copies of an enhancer is known to increase expression in mammalian systems as well (Downes *et al.* 2021). Although a change in enhancer number and position can cause misexpression (Will *et al.* 2017), in some contexts having multiple enhancers can be advantageous as it can provide robustness against disease-causing mutations (Osterwalder *et al.* 2018; Wang and Goldstein 2020). In summary, we show that RET-induced rearrangements can change enhancer copy number and position, fueling *cis*-regulatory variation vital for genomic evolution.

## Data Availability

Maize genetic stocks are available upon request. The authors affirm that all data necessary for confirming the conclusions of the article are present within the article, figures, and tables. Supplementary material is available on figshare: https://doi.org/10.25386/genetics.19944359.

## References

[iyac124-B1] Athma P, Grotewold E, Peterson T. Insertional mutagenesis of the maize *P* gene by intragenic transposition of *Ac*. Genetics. 1992;131(1):199–209. 10.1093/genetics/131.1.199 1317315 PMC1204954

[iyac124-B2] Anderson RE, Brink RA. Kernel pattern in variegated pericarp maize and the frequency of self-colored offspring. Am J Bot. 1952;39(9):637–644. 10.2307/2438369.

[iyac124-B3] Barclay PC, Brink RA. The relation between modulator and activator in maize. Proc Natl Acad Sci U S A. 1954;40(12):1118–1126. 10.1073/pnas.40.12.1118 16589598 PMC1063977

[iyac124-B4] Bartman CR, Hsu SC, Hsiung CC, Raj A, Blobel GA. Enhancer regulation of transcriptional bursting parameters revealed by forced chromatin looping. Mol Cell. 2016;62(2):237–247. 10.1016/j.molcel.2016.03.007 27067601 PMC4842148

[iyac124-B5] Batanian JR, Eswara MS. De novo apparently balanced complex chromosome rearrangement (CCR) involving chromosomes 4, 18, and 21 in a girl with mental retardation: report and review. Am J Med Genet. 1998;78(1):44–51. 10.1002/(SICI)1096-8628(19980616)78:1<44::AID-AJMG9>3.0.CO;2-L 9637422

[iyac124-B6] Bompadre O, Andrey G. Chromatin topology in development and disease. Curr Opin Genet Dev. 2019;55:32–38. 10.1016/j.gde.2019.04.007 31125724

[iyac124-B7] Chakraborty S, Kopitchinski N, Eraso A, Awasthi P, Chari R, Rocha PP. High affinity enhancer-promoter interactions can bypass CTCF/cohesin-mediated insulation and contribute to phenotypic robustness. bioRxiv. 10.1101/2021.12.30.474562, 2022.

[iyac124-B8] Chen J, Greenblatt IM, Dellaporta SL. Transposition of *Ac* from the *P* locus of maize into unreplicated chromosomal sites. Genetics. 1987;117(1):109–116. 10.1093/genetics/117.1.109 2822530 PMC1203179

[iyac124-B9] Dekker J, Marti-Renom MA, Mirny LA. Exploring the three-dimensional organization of genomes: interpreting chromatin interaction data. Nat Rev Genet. 2013;14(6):390–403. 10.1038/nrg3454 23657480 PMC3874835

[iyac124-B10] Deng W, Lee J, Wang H, Miller J, Reik A, Gregory PD, Dean A, Blobel GA. Controlling long-range genomic interactions at a native locus by targeted tethering of a looping factor. Cell. 2012;149(6):1233–1244. 10.1016/j.cell.2012.03.051 22682246 PMC3372860

[iyac124-B11] Doğan ES, Liu C. Three-dimensional chromatin packing and positioning of plant genomes. Nature Plants. 2018;4(8):521–529. 10.1038/s41477-018&ndash;0199-5 30061747

[iyac124-B12] Dooner HK, Belachew A. Chromosome breakage by pairs of closely linked transposable elements of the *Ac-Ds* family in maize. Genetics. 1991;129(3):855–862. 10.1093/genetics/129.3.855 1661257 PMC1204752

[iyac124-B13] Dooner HK, Robbins TP, Jorgensen RA. Genetic and developmental control of anthocyanin biosynthesis. Annu Rev Genet. 1991;25(1):173–199. 10.1146/annurev.ge.25.120191.001133 1839877

[iyac124-B14] Downes DJ, Beagrie RA, Gosden ME, Telenius J, Carpenter SJ, Nussbaum L, De Ornellas S, Sergeant M, Eijsbouts CQ, Schwessinger R, et al High-resolution targeted 3C interrogation of *Cis*-regulatory element organization at genome-wide scale. Nat Commun. 2021;12(1):531. 10.1038/s41467-020&ndash;20809-6 PMC7822813 33483495

[iyac124-B15] Emerson RA. Genetical studies of variegated pericarp in maize. Genetics. 1917;2(1):1–35. 10.1093/genetics/2.1.1 17245873 PMC1193706

[iyac124-B16] Giardino D, Corti C, Ballarati L, Colombo D, Sala E, Villa N, Piombo G, Pierluigi M, Faravelli F, Guerneri S, et al De novo balanced chromosome rearrangements in prenatal diagnosis. Prenat Diagn. 2009;29(3):257–265. 10.1002/pd.2215 19248039

[iyac124-B17] Goettel W, Messing J. Change of gene structure and function by non-homologous end-joining, homologous recombination, and transposition of DNA. PLoS Genet. 2009;5(6):e1000516. 10.1371/journal.pgen.1000516 19521498 PMC2686159

[iyac124-B18] Greenblatt IM, Brink RA. Twin mutations in medium variegated pericarp maize. Genetics. 1962;47(4):489–501. 10.1093/genetics/47.4.489 17248099 PMC1210347

[iyac124-B19] Grotewold E, Drummond BJ, Bowen B, Peterson T. The *Myb*-homologous *P* gene controls phlobaphene pigmentation in maize floral organs by directly activating a flavonoid biosynthetic gene subset. Cell. 1994;76(3):543–553. 10.1016/0092&ndash;8674(94)90117-1 8313474

[iyac124-B20] Heger P, Marin B, Bartkuhn M, Schierenberg E, Wiehe T. The chromatin insulator CTCF and the emergence of metazoan diversity. Proc Natl Acad Sci U S A. 2012;109(43):17507–17512. 10.1073/pnas.1111941109 23045651 PMC3491479

[iyac124-B21] Hu Y, Colantonio V, Müller BSF, Leach KA, Nanni A, Finegan C, Wang B, Baseggio M, Newton CJ, Juhl EM, et al Genome assembly and population genomic analysis provide insights into the evolution of modern sweet corn. Nat Commun. 2021;12:1227. 10.1038/s41467-021&ndash;21380-4 PMC7902669 33623026

[iyac124-B22] Huang JT, Dooner HK. Macrotransposition and other complex chromosomal restructuring in maize by closely linked transposons in direct orientation. Plant Cell. 2008;20(8). 10.1105/tpc.108.060582 PMC2553603 18708475

[iyac124-B23] Kant JA, Fornace AJ, Saxe D, Simon MI, McBride OW, Crabtree GR. Evolution and organization of the fibrinogen locus on chromosome 4: gene duplication accompanied by transposition and inversion. Proc Natl Acad Sci U S A. 1985;82(8):2344–2348. 10.1073/pnas.82.8.2344 2986113 PMC397554

[iyac124-B24] Kramer V, Shaw JR, Senior ML, Hannah LC. The *Sh2-R* allele of the maize *Shrunken-2* locus was caused by a complex chromosomal rearrangement. Theor Appl Genet. 2015;128(3):445–452. 10.1007/s00122-014&ndash;2443-3 25504539

[iyac124-B25] Krumm A, Duan Z. Understanding the 3D genome: emerging impacts on human disease. Semin Cell Dev Biol. 2019;90:62–77. 10.1016/j.semcdb.2018.07.004 29990539 PMC6329682

[iyac124-B26] Kvon EZ, Kazmar T, Stampfel G, Yáñez-Cuna JO, Pagani M, Schernhuber K, Dickson BJ, Stark A. Genome-scale functional characterization of *Drosophila* developmental enhancers in vivo. Nature. 2014;512(7512):91–95. 10.1038/nature13395 24896182

[iyac124-B27] Lechelt C, Peterson T, Laird J, Chen SL, Dellaporta E, Dennis WJ, Peacock P Starlinger. Isolation and molecular analysis of the maize *P* locus. Mol General Genet. 1989;219:225–234. 10.1007/BF00261181 2559311

[iyac124-B28] Li ZT, Jayasankar S, Gray DJ. Bi-directional duplex promoters with duplicated enhancers significantly increase transgene expression in grape and tobacco. Transgenic Res. 2004;13(2):143–154. 10.1023/B:TRAG.0000026074.11859.77 15198202

[iyac124-B29] Lieberman-Aiden E, van Berkum NL, Williams L, Imakaev M, Ragoczy T, Telling A, Amit I, Lajoie BR, Sabo PJ, Dorschner MO, et al Comprehensive mapping of long-range interactions reveals folding principles of the human genome. Science. 2009;326(5950):289–293. 10.1126/science.1181369 19815776 PMC2858594

[iyac124-B30] Louwers M, Bader M, Haring M, van Driel R, de Laat W, Stam M. Tissue- and expression level-specific chromatin looping at maize *B1* epialleles. Plant Cell. 2009;21(3):832–842. 10.1105/tpc.108.064329 19336692 PMC2671708

[iyac124-B31] Louwers M, Splinter E, van Driel R, de Laat W, Stam M. Studying physical chromatin interactions in plants using chromosome conformation capture (3C). Nat Protoc. 2009;4(8):1216–1229. 10.1038/nprot.2009.113 19644461

[iyac124-B32] Lupiáñez DG, Kraft K, Heinrich V, Krawitz P, Brancati F, Klopocki E, Horn D, Kayserili H, Opitz JM, Laxova R, et al Disruptions of topological chromatin domains cause pathogenic rewiring of gene-enhancer interactions. Cell. 2015;161(5):1012–1025. 10.1016/j.cell.2015.04.004 25959774 PMC4791538

[iyac124-B442350996] McVey M, Lee SE. MMEJ repair of double-strand breaks (director's cut): deleted sequences and alternative endings. Trends Genet. 2008;24(11):529–538. 18809224 10.1016/j.tig.2008.08.007 PMC5303623

[iyac124-B33] Moreno MA, Chen J, Greenblatt I, Dellaporta SL. Reconstitutional mutagenesis of the maize *P* gene by short-range *Ac* transpositions. Genetics. 1992;131(4):939–956. 10.1093/genetics/131.4.939 1325389 PMC1205104

[iyac124-B34] Nolis IK, McKay DJ, Mantouvalou E, Lomvardas S, Merika M, Thanos D. Transcription factors mediate long-range enhancer-promoter interactions. Proc Natl Acad Sci U S A. 2009;106(48):20222–20227. 10.1073/pnas.0902454106 19923429 PMC2779200

[iyac124-B35] Nora EP, Dekker J, Heard E. Segmental folding of chromosomes: a basis for structural and regulatory chromosomal neighborhoods? BioEssays. 2013;35(9):818–828. 10.1002/bies.201300040 23832846 PMC3874840

[iyac124-B36] Ochman H, Gerber AS, Hartl DL. Genetic applications of an inverse polymerase chain reaction. Genetics. 1988;120(3):621–623. 10.1093/genetics/120.3.621 2852134 PMC1203539

[iyac124-B37] Osterwalder M, Barozzi I, Tissières V, Fukuda-Yuzawa Y, Mannion BJ, Afzal SY, Lee EA, Zhu Y, Plajzer-Frick I, Pickle CS, et al Enhancer redundancy provides phenotypic robustness in mammalian development. Nature. 2018;554(7691):239–243. 10.1038/nature25461 29420474 PMC5808607

[iyac124-B38] Pellestor F, Anahory T, Lefort G, Puechberty J, Liehr T, Hédon B, Sarda P. Complex chromosomal rearrangements: origin and meiotic behavior. Hum Reprod Update. 2011;17(4):476–494. 10.1093/humupd/dmr010 21486858

[iyac124-B39] Ricci WA, Lu Z, Ji L, Marand AP, Ethridge CL, Murphy NG, Noshay JM, Galli M, Mejía-Guerra MK, Colomé-Tatché M, et al Widespread long-range *Cis*-regulatory elements in the maize genome. Nature Plants. 2019;5(12):1237–1249. 10.1038/s41477-019&ndash;0547-0 31740773 PMC6904520

[iyac124-B40] Saghai-Maroof MA, Soliman KM, Jorgensen RA, Allard RW. Ribosomal DNA Spacer-length polymorphisms in barley: mendelian inheritance, chromosomal location, and population dynamics. Proc Natl Acad Sci U S A. 1984;81(24):8014–8018. 10.1073/pnas.81.24.8014 6096873 PMC392284

[iyac124-B41] Sharma SP, Peterson T. Rapid detection of transposon-induced genome rearrangements. Methods Mol Biol. 2021;2250. 10.1007/978-1-0716-1134-0_13 33900600

[iyac124-B42] Sharma SP, Zuo T, Peterson T. Transposon-induced inversions activate gene expression in the maize pericarp. Genetics. 2021;218(2):iyab062. 10.1093/GENETICS/IYAB062 PMC8225341 33905489

[iyac124-B43] Sidorenko L, Peterson T. Transgene-induced silencing identifies sequences involved in the establishment of paramutation of the maize *P1* gene. Plant Cell. 2001;13(2):319–335. 10.1105/tpc.13.2.319 11226188 PMC102245

[iyac124-B44] Sidorenko L, Li X, Cocciolone SM, Chopra S, Tagliani L, Bowen B, Daniels M, Peterson T. Complex structure of a maize *Myb* gene promoter: functional analysis in transgenic plants. Plant J. 2000;22(6):471–482. 10.1046/j.1365-313X.2000.00750.x 10886767

[iyac124-B45] Sidorenko L, Li X, Tagliani L, Bowen B, Peterson T. Characterization of the regulatory elements of the maize *P-rr* gene by transient expression assays. Plant Mol Biol. 1999;39(1):11–19. 10.1023/A:1006172815663 10080705

[iyac124-B46] Singh M, Lewis PE, Hardeman K, Bai L, Rose JKC, Mazourek M, Chomet P, Brutnell TP. *Activator* mutagenesis of the pink *Scutellum1/Viviparous7* locus of maize. Plant Cell. 2003;15(4):874–884. 10.1105/tpc.010249 12671084 PMC152336

[iyac124-B47] Su W, Sharma SP, Peterson T. Evolutionary impacts of alternative transposition. Origin Evol Biodivers. 2018;113–130. 10.1007/978-3-319-95954-2_7

[iyac124-B48] Su W, Zuo T, Peterson T. Ectopic expression of a maize gene is induced by composite insertions generated through alternative transposition. Genetics. 2020;216(4):1039–1049. 10.1534/genetics.120.303592 32988986 PMC7768264

[iyac124-B49] Tolhuis B, Palstra RJ, Splinter E, Grosveld F, de Laat W. Looping and interaction between hypersensitive sites in the active β-globin locus. Mol Cell. 2002;10(6). 10.1016/S1097-27655 12504019

[iyac124-B50] Vermeulen S, Menten B, van Roy N, van Limbergen H, de Paepe A, Mortier G, Speleman F. Molecular cytogenetic analysis of complex chromosomal rearrangements in patients with mental retardation and congenital malformations: delineation of 7q21.11 breakpoints. Am J Med Genet. 2004;124A(1):10–18. 10.1002/ajmg.a.20378 14679581

[iyac124-B51] Wang D, Yu C, Zhang J, Peterson T. Excision and reinsertion of *Ac macrotransposons* in maize. Genetics. 2022;221(4):iyac067. 10.1093/genetics/iyac067 PMC9339288 35471241

[iyac124-B52] Wang D, Peterson T. Isolation of sequences flanking *Ac* insertion sites by *Ac* casting. Methods Mol Biol. 2013;1057. 10.1007/978-1-62703-568-2_8 23918424

[iyac124-B53] Wang X, Goldstein DB. Enhancer domains predict gene pathogenicity and inform gene discovery in complex disease. Am J Hum Genet. 2020;106(2):215–233. 10.1016/j.ajhg.2020.01.012 PMC7010980 32032514

[iyac124-B54] Weber B, Zicola J, Oka R, Stam M. Plant enhancers: a call for discovery. Trends Plant Sci. 2016;21(11):974–987. 10.1016/j.tplants.2016.07.013 27593567

[iyac124-B55] Will AJ, Cova G, Osterwalder M, Chan W-L, Wittler L, Brieske N, Heinrich V, de Villartay J-P, Vingron M, Klopocki E, et al Composition and dosage of a multipartite enhancer cluster control developmental expression of Ihh (Indian Hedgehog). Nat Genet. 2017;49(10):1539–1545. 10.1038/ng.3939 28846100 PMC5617800

[iyac124-B56] Yu C, Zhang J, Peterson T. Genome rearrangements in maize induced by alternative transposition of reversed *Ac/Ds* termini. Genetics. 2011;188(1):59–67. 10.1534/genetics.111.126847 21339479 PMC3120142

[iyac124-B57] Yu C, Zhang J, Pulletikurti V, Weber DF, Peterson T. Spatial configuration of transposable element *Ac* termini affects their ability to induce chromosomal breakage in maize. Plant Cell. 2010;22(3):744–754. 10.1105/tpc.109.070052 20228246 PMC2861456

[iyac124-B58] Zhang F, Peterson T. Comparisons of maize *Pericarp Color1* alleles reveal paralogous gene recombination and an organ-specific enhancer region. Plant Cell. 2005a;17(3):903–914. 10.1105/tpc.104.029660 15722466 PMC1069707

[iyac124-B59] Zhang J, Peterson T. Transposition of Reversed *Ac* element ends generates chromosome rearrangements in maize. Genetics. 2004;167(4):1929–1937. 10.1534/genetics.103.026229 15342530 PMC1471009

[iyac124-B60] Zhang J, Peterson T. A segmental deletion series generated by sister-chromatid transposition of *Ac* transposable elements in maize. Genetics. 2005b;171(1):333–344. 10.1534/genetics.104.035576 15965263 PMC1456524

[iyac124-B61] Zhang J, Yu C, Pulletikurti V, Lamb J, Danilova T, Weber DF, Birehler J, Peterson T. Alternative *Ac/Ds* transposition induces major chromosomal rearrangements in maize. Genes and Development. 2009;23(6):755–765. 10.1101/gad.1776909 19299561 PMC2661611

[iyac124-B62] Zhang J, Zhang F, Peterson T. Transposition of reversed *Ac* element ends generates novel chimeric genes in maize. PLoS Genet. 2006;2(10):e164. 10.1371/journal.pgen.0020164 17029561 PMC1592236

[iyac124-B63] Zhang J, Zuo T, Peterson T. Generation of tandem direct duplications by reversed-ends transposition of maize *Ac* elements. PLoS Genet. 2013;9(8):e1003691. 10.1371/journal.pgen.1003691 23966872 PMC3744419

[iyac124-B64] Zhang J, Zuo T, Wang D, Peterson T. Transposition-mediated DNA Re-replication in maize. eLife. 2014;3:e03724. 10.7554/eLife.03724 PMC4270019 25406063

[iyac124-B65] Zhang P, Chopra S, Peterson T. A segmental gene duplication generated differentially expressed *Myb*-homologous genes in maize. Plant Cell. 2000;12(12):2311–2322. 10.1105/tpc.12.12.2311 11148280 PMC102220

[iyac124-B66] Zheng L, McMullen MD, Bauer E, Schön C-C, Gierl A, Frey M. Prolonged expression of the *BX1* signature enzyme is associated with a recombination hotspot in the benzoxazinoid gene cluster in *Zea mays*. J Exp Bot. 2015;66(13):3917–3930. 10.1093/jxb/erv192 25969552 PMC4473990

